# Equation of State of Four- and Five-Dimensional Hard-Hypersphere Mixtures

**DOI:** 10.3390/e22040469

**Published:** 2020-04-20

**Authors:** Mariano López de Haro, Andrés Santos, Santos B. Yuste

**Affiliations:** 1 Instituto de Energías Renovables, Universidad Nacional Autónoma de México (U.N.A.M.), Temixco, Morelos 62580, Mexico; malopez@unam.mx; 2 Departamento de Física and Instituto de Computación Científica Avanzada (ICCAEx), Universidad de Extremadura, E-06006 Badajoz, Spain; santos@unex.es

**Keywords:** equation of state, hard hyperspheres, fluid mixtures

## Abstract

New proposals for the equation of state of four- and five-dimensional hard-hypersphere mixtures in terms of the equation of state of the corresponding monocomponent hard-hypersphere fluid are introduced. Such proposals (which are constructed in such a way so as to yield the exact third virial coefficient) extend, on the one hand, recent similar formulations for hard-disk and (three-dimensional) hard-sphere mixtures and, on the other hand, two of our previous proposals also linking the mixture equation of state and the one of the monocomponent fluid but unable to reproduce the exact third virial coefficient. The old and new proposals are tested by comparison with published molecular dynamics and Monte Carlo simulation results and their relative merit is evaluated.

## 1. Introduction

The interest in studying systems of *d*-dimensional hard spheres has been present for many decades and still continues to stimulate intensive research [1,2,3,4,5,6,7,8,9,10,11,12,13,14,15,16,17,18,19,20,21,22,23,24,25,26,27,28,29,30,31,32,33,34,35,36,37,38,39,40,41,42,43,44,45,46,47,48,49,50,51,52,53,54,55,56,57,58,59,60,61,62,63,64,65,66,67,68,69,70,71,72,73,74,75,76,77,78,79,80,81,82,83,84,85,86,87,88,89,90,91,92,93,94,95,96]. This interest is based on the versatility of such systems that allows one to gain insight into, among other things, the equilibrium and dynamical properties of simple fluids, colloids, granular matter, and glasses with which they share similar phenomenology. For instance, it is well known that all *d*-dimensional hard-sphere systems undergo a fluid-solid phase transition which occurs at smaller packing fractions as the spatial dimension is increased. This implies that mean-field-like descriptions of this transition become mathematically simpler and more accurate as one increases the number of dimensions. Additionally, in the limit of infinite dimension one may even derive analytical results for the thermodynamics, structure, and phase transitions of such hypersphere fluids [1,2,3,4,5,6,7,8,9,10,11,12,13]. In particular, the equation of state (EOS) truncated at the level of the second virial coefficient becomes exact in this limit [8].

While of course real experiments cannot be performed in these systems, they are amenable to computer simulations and theoretical developments. Many aspects concerning hard hyperspheres have been already dealt with, such as thermodynamic and structural properties [13,14,15,16,17,18,19,20,21,22,23,24,25,26,27,28,29,30,31,32,33,34,35,36,37,38,39,40,41,42,43,44,45,46,47,48,49,50,51,52,53,54,55,56,57,58,59,60,61,62,63,64,65,66,67], virial coefficients [67,68,69,70,71,72,73,74,75,76,77,78,79,80], and disordered packings [52,81,82,83,84,85,86,87,88,89,90,91] or glassy behavior [12,81,82,92]. Nevertheless, due to the fact that (except in the infinite dimensional case) no exact analytical results are available, efforts to clarify or reinforce theoretical developments are worth pursuing. In the case of mixtures of hard hyperspheres this is particularly important since, comparatively speaking, the literature pertaining to them is not very abundant. To the best of our knowledge, the first paper reporting an (approximate) EOS for additive binary hard-hypersphere fluid mixtures is the one by González et al. [28], in which they used the overlap volume approach. What they did was to compute the partial direct correlation functions through an interpolation between the exact low-density and the Percus–Yevick high-density behavior of such functions to produce a Carnahan–Starling-like EOS which they subsequently compared with the (very few then) available simulation data for additive hard-disk mixtures. A few years later, we [32,48] proposed an ansatz for the contact values of the partial radial distribution functions complying with some exact limiting conditions to derive an EOS (henceforth denoted with the label “e1”) of a multicomponent *d*-dimensional hard-sphere fluid in terms of the one of the single monocomponent system. To our knowledge, the first simulation results for the structural and thermodynamic properties of additive hard-hypersphere mixtures were obtained via molecular dynamics (MD) for a few binary mixtures in four and five spatial dimensions by González-Melchor et al. [36], later confirmed by Monte Carlo (MC) computations by Bishop and Whitlock [41]. The comparison between such simulation results and our e1 EOS [32] led to very reasonable agreement. Later, we proposed a closely related EOS (henceforth denoted with the label “e2”) stemming from additional exact limiting conditions applied to the contact values of the partial radial distribution functions [37,48]. A limitation of these proposals is that, except in the three-dimensional case, they are unable to yield the exact third virial coefficient. As shown below, extensions of these EOS (denoted as “$\bar{e}$1” and “$\bar{e}$2”) complying with the requirement that the third virial coefficient computed from them is the exact one, may be introduced with little difficulty. More recently, we have developed yet another approximate EOS (henceforth denoted with the label “sp”) for *d*-dimensional hard-sphere fluid mixtures [63,64,93], and newer simulation results for hard hypersphere mixtures have also been obtained [57,58,59]. It is the aim of this paper to carry out a comparison between available simulation data for binary additive four- and five-dimensional hypersphere fluid mixtures and our theoretical proposals.

The paper is organized as follows. In order to make it self-contained, in Section 2 we provide a brief outline of the approaches we have followed to link the EOS of a polydisperse *d*-dimensional hard-sphere mixture and that of the corresponding monocomponent system. Section 3 presents the specific cases of four and five spatial dimensions, the choice of the EOS of the monocomponent system to complete the mapping, and the comparison with the simulation data. We close the paper in Section 4 with a discussion of the results and some concluding remarks.

## 2. Mappings between the Equation of State of the Polydisperse Mixture and That of the Monocomponent System

Let us begin by considering a mixture of additive hard spheres in *d* dimensions with an arbitrary number *s* of components. This number *s* may even be infinite, i.e., the system may also be a polydisperse mixture with a continuous size distribution. The additive hard core of the interaction between a sphere of species *i* and a sphere of species *j* is $\sigma_{ij}=\frac{1}{2}\left(\sigma_{i}+\sigma_{j}\right)$, where the diameter of a sphere of species *i* is $\sigma_{ii}=\sigma_{i}$. Let the number density of the mixture be ρ and the mole fraction of species *i* be $x_{i}=\rho_{i}/\rho$, where $\rho_{i}$ is the number density of species *i*. In terms of these quantities, the packing fraction is given by $\eta =v_{d}\rho M_{d}$, where $v_{d}={\left(\pi /4\right)}^{d/2}/\Gamma \left(1+d/2\right)$ is the volume of a *d*-dimensional sphere of unit diameter, Γ(·) is the Gamma function, and $M_{n}\equiv \langle \sigma^{n}\rangle =\sum_{i=1}^{s}x_{i}\sigma_{i}^{n}$ denotes the *n*th moment of the diameter distribution.

Unfortunately, no exact explicit EOS for a fluid mixture of *d*-dimensional hard spheres is available. The (formal) virial expression for such EOS involves only the contact values $g_{ij}\left(\sigma_{ij}^{+}\right)$ of the radial distribution functions $g_{ij}\left(r\right)$, where *r* is the distance, namely

(1) $$Z\left(\eta \right)=1+\frac{2^{d-1}}{M_{d}}\eta \sum_{i,j=1}^{s}x_{i}x_{j}\sigma_{ij}^{d}g_{ij}\left(\sigma_{ij}^{+}\right),$$

where $Z=p/\rho k_{B}T$ is the compressibility factor of the mixture, *p* being the pressure, $k_{B}$ the Boltzmann constant, and *T* the absolute temperature. Hence, a useful way to obtain approximate expressions for the EOS of the mixture is to propose or derive approximate expressions for the contact values $g_{ij}\left(\sigma_{ij}^{+}\right)$. We have already followed this route and the outcome is briefly described in Section 2.1 and Section 2.2. More details may be found in Ref. [48] and references therein.

### 2.1. The e1 Approximation

The basic assumption is that, at a given packing fraction η, the dependence of $g_{ij}\left(\sigma_{ij}^{+}\right)$ on the sets of $\left\{\sigma_{k}\right\}$ and $\left\{x_{k}\right\}$ takes place *only* through the scaled quantity

(2) $$z_{ij}\equiv \frac{\sigma_{i}\sigma_{j}}{\sigma_{ij}}\frac{M_{d-1}}{M_{d}},$$

which we express as

(3) $$g_{ij}\left(\sigma_{ij}^{+}\right)=G\left(\eta ,z_{ij}\right),$$

where the function G(η,z) is *universal*, i.e., it is a common function for all the pairs (i,j), regardless of the composition and number of components of the mixture. Next, making use of some consistency conditions, we have derived two approximate expressions for the EOS of the mixture. The first one, labeled “e1,” indicating that (i) the contact values $g_{ij}\left(\sigma_{ij}^{+}\right)$ used are an *extension* of the monocomponent fluid contact value $g_{s}\equiv g\left(\sigma^{+}\right)$ and that (ii) G(η,z) is a *linear* polynomial in *z*, leads to an EOS that exhibits an excellent agreement with simulations in 2, 3, 4, and 5 dimensions, provided that an accurate $g_{s}$ is used as input [32,36,57,59,67]. This EOS may be written as

(4) $$Z_{e1}\left(\eta \right)=1+\frac{\eta }{1-\eta }2^{d-1}\left(\Omega_{0}-\Omega_{1}\right)+\left[Z_{s}\left(\eta \right)-1\right]\Omega_{1},$$

where the coefficients $\Omega_{m}$ depend only on the composition of the mixture and are defined by

(5) Ωm=2−(d−m)Md−1mMdm+1∑n=0d−md−mnMn+mMd−n.

It is interesting to point out that from Equation (4) one may write the virial coefficients of the mixture $B_{n}$, defined by

(6) $$Z\left(\rho \right)=1+\sum_{n=1}^{\infty }B_{n+1}\rho^{n},$$

in terms of the (reduced) virial coefficients of the single component fluid $b_{n}$ defined by

(7) $$Z_{s}\left(\eta \right)=1+\sum_{n=1}^{\infty }b_{n+1}\eta^{n}.$$

The result is

(8) $${\bar{B}}_{n}^{e1}=\Omega_{1}b_{n}+2^{d-1}\left(\Omega_{0}-\Omega_{1}\right),$$

where ${\bar{B}}_{n}\equiv B_{n}/{\left(v_{d}M_{d}\right)}^{n-1}$ are reduced virial coefficients. Since $b_{2}=2^{d-1}$, Equation (8) yields the *exact* second virial coefficient [63]

(9) $${\bar{B}}_{2}=2^{d-1}\Omega_{0}.$$

In general, however, ${\bar{B}}_{n}^{e1}$ with n≥3 are only approximate. In particular,

(10a) $${\bar{B}}_{3}^{e1}=1+\left(\frac{b_{3}}{4}+2\right)\frac{M_{1}M_{3}}{M_{4}}+3\frac{M_{2}^{2}}{M_{4}}+\left(\frac{3b_{3}}{4}-6\right)\frac{M_{2}M_{3}^{2}}{M_{4}^{2}},\;\left(d=4\right),$$

(10b) $${\bar{B}}_{3}^{e1}=1+\frac{65}{4}\frac{M_{1}M_{4}}{M_{5}}+10\frac{M_{2}M_{3}}{M_{5}}+45\frac{M_{2}M_{4}^{2}}{M_{5}^{2}}+\frac{135}{4}\frac{M_{3}^{2}M_{4}}{M_{5}^{2}},\;\left(d=5\right).$$

In Equation (10a),

(11) $$b_{3}=64\left(\frac{4}{3}-\frac{3\sqrt{3}}{2\pi }\right),\;\left(d=4\right),$$

is the reduced third virial coefficient of a monocomponent four-dimensional fluid, while in Equation (10b) we have taken into account that $b_{3}=106$ if d=5.

It is interesting to note that, by eliminating $\Omega_{0}$ and $\Omega_{1}$ in favor of ${\bar{B}}_{2}$ and ${\bar{B}}_{3}^{e1}$, Equation (4) can be rewritten as

(12) $$Z_{e1}\left(\eta \right)=1+\frac{\eta }{1-\eta }\frac{b_{3}{\bar{B}}_{2}-b_{2}{\bar{B}}_{3}^{e1}}{b_{3}-b_{2}}+\left[Z_{s}\left(\eta \right)-1\right]\frac{{\bar{B}}_{3}^{e1}-{\bar{B}}_{2}}{b_{3}-b_{2}}.$$

### 2.2. The e2 Approximation

The second approximation, labeled “e2,” similarly indicates that (i) the resulting contact values represent an *extension* of the single component contact value $g_{s}$ and that (ii) G(η,z) is a *quadratic* polynomial in *z*. In this case, one also gets a closed expression for the compressibility factor in terms of the packing fraction η and the first few moments $M_{n}$, n≤d. Such an expression is

(13) $$Z_{e2}\left(\eta \right)=Z_{e1}\left(\eta \right)-\left(\Omega_{2}-\Omega_{1}\right)\left[Z_{s}\left(\eta \right)\left(1-2^{d-2}\eta \right)-1-2^{d-2}\frac{\eta }{1-\eta }\right].$$

The associated (reduced) virial coefficients are

(14) $${\bar{B}}_{n}^{e2}={\bar{B}}_{n}^{e1}-\left(\Omega_{2}-\Omega_{1}\right)\left[b_{n}-2^{d-2}\left(1+b_{n-1}\right)\right].$$

Again, since $b_{1}=1$ and $b_{2}=2^{d-1}$, the exact second virial coefficient, Equation (9), is recovered for any dimensionality. Additionally, in the case of spheres (d=3), $b_{3}=10$ and thus ${\bar{B}}_{3}^{e1}={\bar{B}}_{3}^{e2}=4\Omega_{0}+6\Omega_{1}$, which is the exact result for that dimensionality. In the cases of d=4 and d=5, one has

(15a) $${\bar{B}}_{3}^{e2}=1+\left(\frac{b_{3}}{2}-7\right)\frac{M_{1}M_{3}}{M_{4}}+3\frac{M_{2}^{2}}{M_{4}}+\left(b_{3}-15\right)\frac{M_{2}M_{3}^{2}}{M_{4}^{2}}+\left(18-\frac{b_{3}}{2}\right)\frac{M_{3}^{4}}{M_{4}^{3}},\;\left(d=4\right),$$

(15b) $${\bar{B}}_{3}^{e2}=1+\frac{25}{2}\frac{M_{1}M_{4}}{M_{5}}+10\frac{M_{2}M_{3}}{M_{5}}+\frac{75}{2}\frac{M_{2}M_{4}^{2}}{M_{5}^{2}}+\frac{45}{2}\frac{M_{3}^{2}M_{4}}{M_{5}^{2}}+\frac{45}{2}\frac{M_{3}M_{4}^{3}}{M_{5}^{3}},\;\left(d=5\right).$$

It is also worthwhile noting that $\Omega_{1}=\Omega_{2}$ in the case of disks (d=2) and thus $Z_{e1}\left(\eta \right)=Z_{e2}\left(\eta \right)$ for those systems.

### 2.3. Exact Third Virial Coefficient. Modified Versions of the e1 and e2 Approximations

As said above, both ${\bar{B}}_{3}^{e1}$ and ${\bar{B}}_{3}^{e2}$ differ from the exact third virial coefficient, except in the three-dimensional case (d=3). The exact expression is [63]

(16a) $${\bar{B}}_{3}=\frac{1}{M_{d}^{2}}\sum_{i,j,k=1}^{s}x_{i}x_{j}x_{k}{\hat{B}}_{ijk},$$

(16b) $${\hat{B}}_{ijk}=\frac{d^{2}}{3}2^{5d/2-1}\Gamma \left(d/2\right){\left(\sigma_{ij}\sigma_{ik}\sigma_{jk}\right)}^{d/2}\int_{0}^{\infty }\frac{d\kappa }{\kappa^{1+d/2}}J_{d/2}\left(\kappa \sigma_{ij}\right)J_{d/2}\left(\kappa \sigma_{ik}\right)J_{d/2}\left(\kappa \sigma_{jk}\right),$$

where $J_{n}\left(\cdot \right)$ is the Bessel function of the first kind of order *n*.

For odd dimensionality, it turns out that the composition-independent coefficients ${\hat{B}}_{ijk}$ have a polynomial dependence on $\sigma_{i}$, $\sigma_{j}$, and $\sigma_{k}$. As a consequence, the third virial coefficient ${\bar{B}}_{3}$ can be expressed in terms of moments $M_{n}$ with 1≤n≤d. In particular [63],

(17) $${\bar{B}}_{3}=1+10\frac{M_{1}M_{4}}{M_{5}}+20\frac{M_{2}M_{3}}{M_{5}}+25\frac{M_{2}M_{4}^{2}}{M_{5}^{2}}+50\frac{M_{3}^{2}M_{4}}{M_{5}^{2}},\;\left(d=5\right).$$

On the other hand, for even dimensionality the dependence of ${\hat{B}}_{ijk}$ on $\sigma_{i}$, $\sigma_{j}$, and $\sigma_{k}$ is more complex than polynomial. In particular, for a binary mixture (s=2) with d=4 one has

(18a) $${\hat{B}}_{111}=b_{3}\sigma_{1}^{8},\;\left(d=4\right),$$

(18b) $$\begin{array}{cc} {\hat{B}}_{112}= & \sigma_{1}^{8}\frac{16{\left(1+q\right)}^{4}}{3}[1-\frac{1}{8\pi }\left(1-q\right)\left(3+q\right)\left(5+2q+q^{2}\right)\arcsin\frac{1}{1+q}-\frac{\sqrt{q(2+q)}}{24\pi {\left(1+q\right)}^{4}}\left(45+138q\right.\\ & \left.+113q^{2}+68q^{3}+47q^{4}+18q^{5}+3q^{6}\right)],\;\left(d=4\right), \end{array}$$

where $q\equiv \sigma_{2}/\sigma_{1}$ is the size ratio. The expressions for ${\hat{B}}_{222}$ and ${\hat{B}}_{122}$ can be obtained from Equations (18a) and (18b), respectively, by the replacements $\sigma_{1}\to \sigma_{2}$, $q\to q^{-1}$.

**Figure 1:**
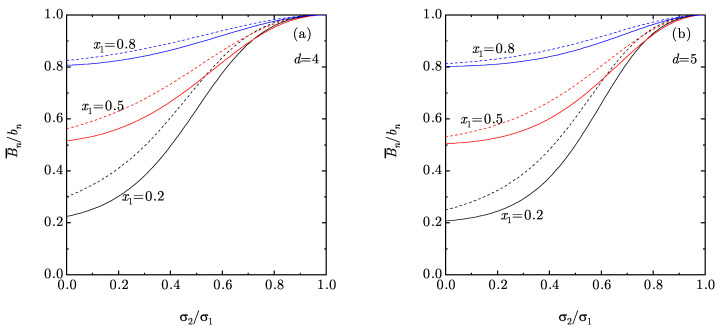
Plot of the ratios ${\bar{B}}_{2}/b_{2}$ (dashed lines) and ${\bar{B}}_{3}/b_{3}$ (solid lines) vs. the size ratio $\sigma_{2}/\sigma_{1}$ for binary mixtures with mole fractions $x_{1}=0.2$, 0.5, and 0.8. Panel (**a**) corresponds to d=4, while panel (**b**) corresponds to d=5.

Figure 1 displays the size-ratio dependence of the exact second and third virial coefficients for three representative binary compositions of four- and five-dimensional systems. The degree of bidispersity of a certain binary mixture can be measured by the distances $1-{\bar{B}}_{2}/b_{2}$ and $1-{\bar{B}}_{3}/b_{3}$. In this sense, Figure 1 shows that, as expected, the degree of bidispersity grows monotonically as the small-to-big size ratio decreases at a given mole fraction. It also increases as the concentration of the big spheres decreases at a given size ratio, except if the latter ratio is close enough to unity.

**Figure 2:**
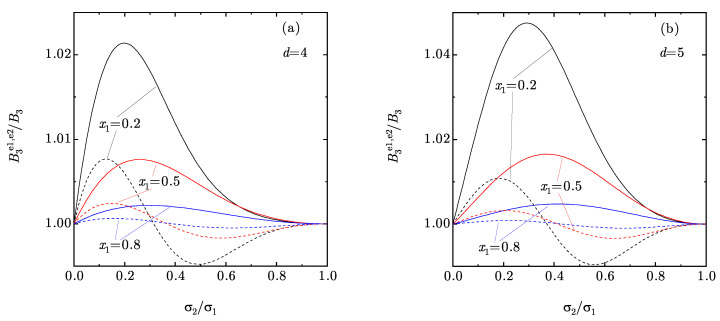
Plot of the ratios $B_{3}^{e1}/B_{3}$ (solid lines) and $B_{3}^{e2}/B_{3}$ (dashed lines) vs. the size ratio $\sigma_{2}/\sigma_{1}$ for binary mixtures with mole fractions $x_{1}=0.2$, 0.5, and 0.8. Panel (**a**) corresponds to d=4, while panel (**b**) corresponds to d=5.

To assess the quality of the approximate coefficients (10) and (15), we plot in Figure 2 the ratios $B_{3}^{e1}/B_{3}$ and $B_{3}^{e2}/B_{3}$ as functions of the size ratio $\sigma_{2}/\sigma_{1}$ for the same three representative binary compositions as in Figure 1. As we can observe, both the e1 and e2 approximations predict values for the third virial coefficient in overall good agreement with the exact values, especially as the concentration of the big spheres increases. The e1 approximation overestimates $B_{3}$ and generally performs worse than the e2 approximation, which tends to overestimate (underestimate) $B_{3}$ if the concentration of the big spheres is sufficiently small (large). Additionally, the agreement is better in the four-dimensional case than for five-dimensional hyperspheres. The latter point is relevant because, as said before, the exact expressions of $B_{3}$ for d=4 are relatively involved [see Equations (18) in the binary case], whereas $B_{3}^{e1}$ and $B_{3}^{e2}$ are just simple combinations of moments [see Equations (10a) and (15a)].

The structure of Equation (12) suggests the introduction of a *modified* version (henceforth labeled as “$\bar{e}$1”) of the e1 EOS by replacing the approximate third virial coefficient ${\bar{B}}_{3}^{e1}$ by the exact one. More specifically,

(19) $$Z_{\bar{e}1}\left(\eta \right)=Z_{e1}\left(\eta \right)+\frac{{\bar{B}}_{3}-{\bar{B}}_{3}^{e1}}{b_{3}-b_{2}}\left[Z_{s}\left(\eta \right)-1-b_{2}\frac{\eta }{1-\eta }\right].$$

Analogously, we introduce the modified version (“$\bar{e}$2”) of the e2 approximation as

(20) $$Z_{\bar{e}2}\left(\eta \right)=Z_{e2}\left(\eta \right)+\frac{{\bar{B}}_{3}-{\bar{B}}_{3}^{e2}}{b_{3}-b_{2}}\left[Z_{s}\left(\eta \right)-1-b_{2}\frac{\eta }{1-\eta }\right].$$

By construction, both $Z_{\bar{e}1}\left(\eta \right)$ and $Z_{\bar{e}2}\left(\eta \right)$ are consistent with the exact second and third virial coefficients. Moreover, $Z_{\bar{e}1}\left(\eta \right)=Z_{\bar{e}2}\left(\eta \right)$ for d=2, while $Z_{\bar{e}1}\left(\eta \right)=Z_{e1}\left(\eta \right)$ and $Z_{\bar{e}2}\left(\eta \right)=Z_{e2}\left(\eta \right)$ for d=3.

### 2.4. The sp Approximation

Additionally, in previous work [63,64,93], we have adopted an approach to relate the EOS of the polydisperse mixture of *d*-dimensional hard spheres to the one of the monocomponent fluid which differs from the e1 and e2 approaches in that it does not make use of Equation (1). This involves expressing the excess free energy per particle ($a^{\mathrm{ex}}$) of a polydisperse mixture of packing fraction η in terms of the one of the corresponding monocomponent fluid ($a_{s}^{\mathrm{ex}}$) of an effective packing fraction $\eta_{\mathrm{eff}}$ as

(21) $$\frac{a^{\mathrm{ex}}\left(\eta \right)}{k_{B}T}+\ln\left(1-\eta \right)=\frac{\alpha }{\lambda }\left[\frac{a_{s}^{\mathrm{ex}}\left(\eta_{\mathrm{eff}}\right)}{k_{B}T}+\ln\left(1-\eta_{\mathrm{eff}}\right)\right].$$

In Equation (21), $\eta_{\mathrm{eff}}$ and η are related through

(22) $$\frac{\eta_{\mathrm{eff}}}{1-\eta_{\mathrm{eff}}}=\frac{1}{\lambda }\frac{\eta }{1-\eta },\;\eta_{\mathrm{eff}}={\left[1+\lambda \left(\eta^{-1}-1\right)\right]}^{-1},$$

while the parameters λ and α are determined by imposing consistency with the (exact) second and third virial coefficients of the mixture, Equations (9) and (16). More specifically [63,64],

(23) $$\lambda =\frac{{\bar{B}}_{2}-1}{b_{2}-1}\frac{b_{3}-2b_{2}+1}{{\bar{B}}_{3}-2{\bar{B}}_{2}+1},\;\alpha =\lambda^{2}\frac{{\bar{B}}_{2}-1}{b_{2}-1}.$$

Note that the ratio η/(1−η) represents a rescaled packing fraction, i.e., the ratio between the volume occupied by the spheres and the remaining void volume. Thus, according to Equation (22), the effective monocomponent fluid associated with a given mixture has a rescaled packing fraction $\eta_{\mathrm{eff}}/\left(1-\eta_{\mathrm{eff}}\right)$ that is λ times smaller than that of the mixture. Moreover, in the case of three-dimensional hard-sphere mixtures, Equations (21)–(23) can be derived in the context of consistent fundamental-measure theories [63,64,97,98].

Taking into account the thermodynamic relation

(24) $$Z\left(\eta \right)=1+\eta \frac{\partial a^{\mathrm{ex}}\left(\eta \right)/k_{B}T}{\partial \eta },$$

the mapping between the compressibility factor of the *d*-dimensional monocomponent system ($Z_{s}$) and the approximate one of the polydisperse mixture that is then obtained from Equation (21) may be expressed as

(25) $$\eta Z_{\mathrm{sp}}\left(\eta \right)-\frac{\eta }{1-\eta }=\alpha \left[\eta_{\mathrm{eff}}Z_{s}\left(\eta_{\mathrm{eff}}\right)-\frac{\eta_{\mathrm{eff}}}{1-\eta_{\mathrm{eff}}}\right],$$

where a label “sp”, motivated by the nomenclature already introduced in connection with the “surplus” pressure ηZ(η)−η/(1−η) [63], has been added to distinguish this compressibility factor from the previous approximations.

Equation (25) shares with Equations (19) and (20) the consistency with the exact second and third virial coefficients. On the other hand, while $Z_{\bar{e}1}\left(\eta \right)$ and $Z_{\bar{e}2}\left(\eta \right)$ are related to the monocomponent compressibility factor $Z_{s}\left(\eta \right)$ evaluated at the same packing fraction η as that of the mixture, $Z_{\mathrm{sp}}\left(\eta \right)$ is related to $Z_{s}\left(\eta_{\mathrm{eff}}\right)$ evaluated at a different (effective) packing fraction $\eta_{\mathrm{eff}}$.

**Figure 3:**
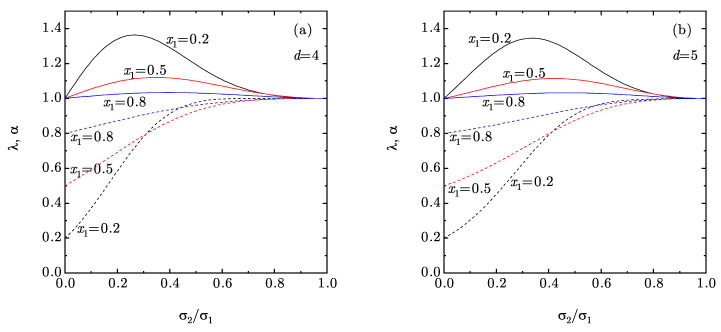
Plot of the coefficients λ (solid lines) and α (dashed lines) [see Equation (23)] vs. the size ratio $\sigma_{2}/\sigma_{1}$ for binary mixtures with mole fractions $x_{1}=0.2$, 0.5, and 0.8. Panel (**a**) corresponds to d=4, while panel (**b**) corresponds to d=5.

Figure 3 shows that λ>1, while α<1, except if the mole fraction of the big spheres is large enough (not shown). According to Equations (22) and (25), this implies that (i) $\eta_{\mathrm{eff}}<\eta$ and (ii) the surplus pressure of the mixture at a packing fraction η is generally smaller than that of the monocomponent fluid at the equivalent packing fraction $\eta_{\mathrm{eff}}$. It is also worthwhile noting that, in contrast to what happens with ${\bar{B}}_{2}$ and ${\bar{B}}_{3}$ (see Figure 1), λ has a nonmonotonic dependence on the size ratio and α also exhibits a nonmonotonic behavior if $x_{1}$ is small enough.

While we have proved the sp approach to be successful for both hard-disk (d=2) [64] and hard-sphere (d=3) [93] mixtures, one of our goals is to test it for d=4 and d=5 as well.

## 3. Comparison with Computer Simulation Results

In order to obtain explicit numerical results for the different approximations to the EOS of four- and five-dimensional hard-sphere mixtures, we require an expression for $Z_{s}\left(\eta \right)$. While other choices are available, we considered here the empirical proposal that works for both dimensionalities by Luban and Michels (LM) [25], which reads

(26) $$Z_{s}\left(\eta \right)=1+b_{2}\eta \frac{1+\left[b_{3}/b_{2}-\zeta \left(\eta \right)b_{4}/b_{3}\right]\eta }{1-\zeta \left(\eta \right)\left(b_{4}/b_{3}\right)\eta +\left[\zeta (\eta )-1\right]\left(b_{4}/b_{2}\right)\eta^{2}},$$

where $\zeta \left(\eta \right)=\zeta_{0}+\zeta_{1}\eta /\eta_{\mathrm{cp}}$, $\eta_{\mathrm{cp}}$ being the crystalline close-packing value. The values of $b_{2}$, $b_{3}$, $b_{4}$, $\zeta_{0}$, $\zeta_{1}$, and $\eta_{\mathrm{cp}}$ are given in Table 1.

In Table 2 we list the systems whose compressibility factor has been obtained from simulation, either using MD [36] or MC [57,59] methods. The values of the corresponding coefficients ${\bar{B}}_{2}$ [see Equation (9)], ${\bar{B}}_{3}$ [see Equations (16)–(18)], λ, and α [see Equation (23)] are also included. We assigned a three-character label to each system, where the first (capital) letter denotes the size ratio (A–F for $\sigma_{2}/\sigma_{1}=\frac{1}{4}$, $\frac{1}{3}$, $\frac{2}{5}$, $\frac{1}{2}$, $\frac{3}{5}$, and $\frac{3}{4}$, respectively), the second (lower-case) letter denotes the mole fraction (a, b, and c for $x_{1}=0.25$, 0.50, and 0.75, respectively), and the digit (4 or 5) denotes the dimensionality.

If, as before, the degree of bidispersity is measured by $1-{\bar{B}}_{2}/b_{2}$ and $1-{\bar{B}}_{3}/b_{3}$, we can observe the following ordering of decreasing bidispersity in the four-dimensional systems: Aa, Ba, Ab, Bb, Da, Cb, Db, Ac, Bc, Eb, Dc, Fa, Fb, and Fc. The same ordering applies in the case of the five-dimensional systems, except that, apart from the absence of the system Eb, the sequence {Ab, Bb, Da} is replaced by either {Ab, Da, Bb} or by {Da, Ab, Bb} if either $1-{\bar{B}}_{2}/b_{2}$ or $1-{\bar{B}}_{3}/b_{3}$ are used, respectively.

It should be stressed that the proposals implied by Equations (4), (13), (19), (20), and (25) may be interpreted in two directions. On the one hand, if $Z_{s}$ is known as a function of the packing fraction, then one can readily compute the compressibility factor of the mixture for any packing fraction and composition [$\eta_{\mathrm{eff}}$ and η being related through Equation (22) in the case of $Z_{\mathrm{sp}}$]; this is the standard view. On the other hand, if simulation data for the EOS of the mixture are available for different densities, size ratios, and mole fractions, Equations (4), (13), (19), (20), and (25) can be used to *infer* the compressibility factor of the monocomponent fluid. This is particularly important in the high-density region, where obtaining data from simulation may be accessible in the case of mixtures but either difficult or not feasible in the case of the monocomponent fluid, as happens in the metastable fluid branch [64,93].

**Figure 4:**
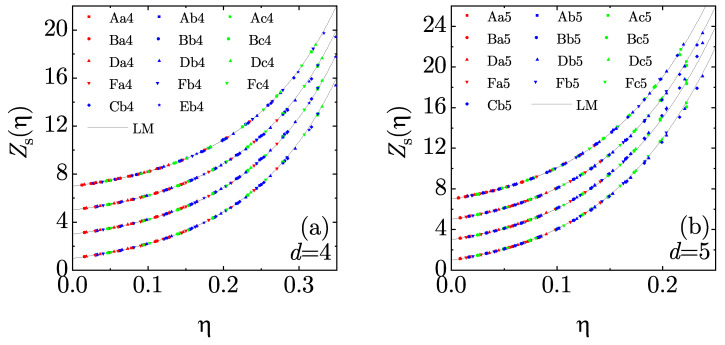
Plot of the monocomponent compressibility factor $Z_{s}\left(\eta \right)$, as inferred from simulation data for the mixtures described in Table 2, according to the theories (from bottom to top) e1, e2, $\bar{e}$1, and sp (the three latter have been shifted vertically for better clarity). The solid lines represent the Luban and Michels (LM) equation of state (EOS), Equation (26). Panel (**a**) corresponds to d=4, while panel (**b**) corresponds to d=5.

In principle, simulation data for different mixtures would yield different inferred functions $Z_{s}\left(\eta \right)$. Thus, without having to use an externally imposed monocomponent EOS, the degree of collapse of the mapping from mixture compressibility factors onto a *common* function $Z_{s}\left(\eta \right)$ is an efficient way of assessing the performance of Equations (4), (13), (19), (20), and (25). As shown in Figure 4, the usefulness of those mappings is confirmed by the nice collapse obtained for all the points corresponding to the mixtures described in Table 2. The inferred data associated with $Z_{\bar{e}2}$ are almost identical to those associated with $Z_{e2}$ and thus they are omitted in Figure 4. Figure 4 also shows that the inferred curves are very close to the LM (monocomponent) EOS, Equation (26), what validates its choice as an accurate function $Z_{s}\left(\eta \right)$ in what follows. Notwithstanding this, one can observe in the high-density regime that the values inferred from simulation data via $Z_{e1}$ and $Z_{\bar{e}1}$ tend to underestimate the LM curve for both d=4 and d=5, while the values inferred via $Z_{e2}$ tend to overestimate it for d=5. Overall, one can say that the best agreement with the LM EOS is obtained by using $Z_{e2}$ and $Z_{\mathrm{sp}}$ for d=4 and d=5, respectively.

Now we turn to a more a direct comparison between the simulation data and the approximate EOS for mixtures. As expected from the indirect representation of Figure 4, we observed a very good agreement (not shown) between the simulation data for the systems displayed in Table 2 and the theoretical predictions obtained from Equations (4), (13), (19), (20), and (25), supplemented by Equation (26).

**Figure 5:**
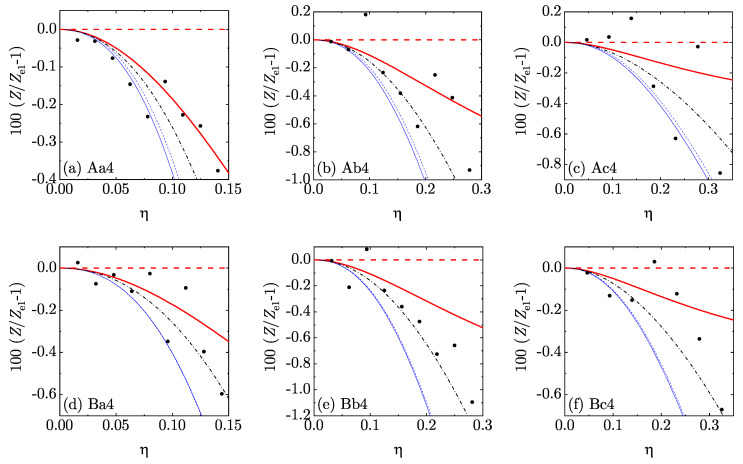
Plot of the relative deviations $100[Z\left(\eta \right)/Z_{e1}\left(\eta \right)-1]$ from the theoretical EOS $Z_{e1}\left(\eta \right)$ for the four-dimensional mixtures Aa4–Bc4 (see Table 2). Thick (red) dashed lines: e1; thick (red) solid lines: $\bar{e}$1; thin (blue) dashed lines: e2; thin (blue) solid lines: $\bar{e}$2; dash-dotted (black) lines: sp; filled (black) circles: MD.

**Figure 6:**
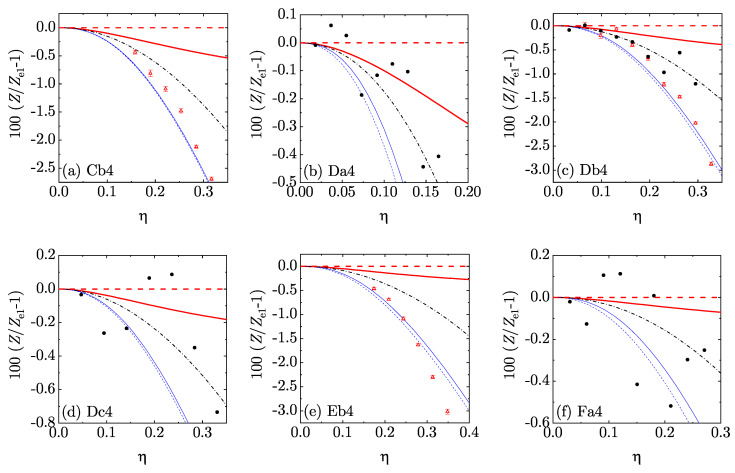

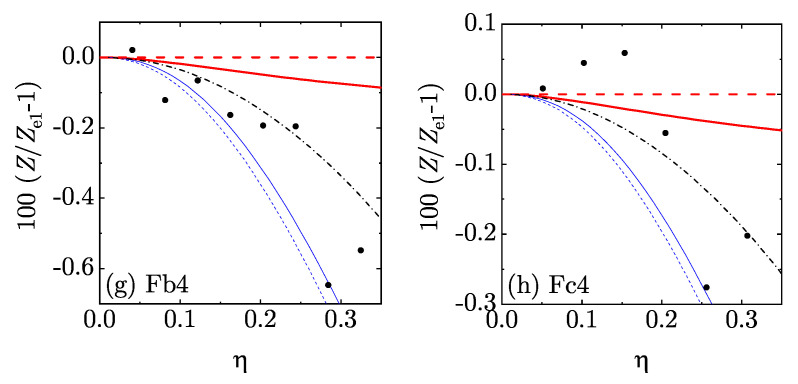
Plot of the relative deviations $100[Z\left(\eta \right)/Z_{e1}\left(\eta \right)-1]$ from the theoretical EOS $Z_{e1}\left(\eta \right)$ for the four-dimensional mixtures Cb4–Fc4 (see Table 2). Thick (red) dashed lines: e1; thick (red) solid lines: $\bar{e}$1; thin (blue) dashed lines: e2; thin (blue) solid lines: $\bar{e}$2; dash-dotted (black) lines: sp; filled (black) circles: MD; open (red) triangles with error bars in panels (**a**,**c**,**e**): MC.

**Figure 7:**
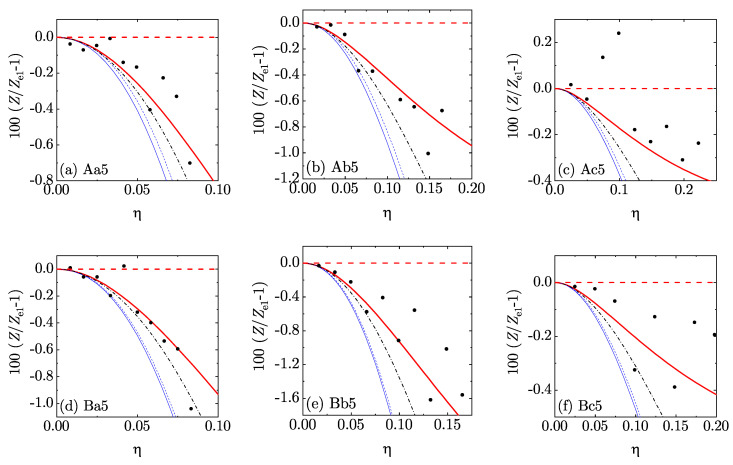
Plot of the relative deviations $100[Z\left(\eta \right)/Z_{e1}\left(\eta \right)-1]$ from the theoretical EOS $Z_{e1}\left(\eta \right)$ for the five-dimensional mixtures Aa5–Bc5 (see Table 2). Thick (red) dashed lines: e1; thick (red) solid lines: $\bar{e}$1; thin (blue) dashed lines: e2; thin (blue) solid lines: $\bar{e}$2; dash-dotted (black) lines: sp; filled (black) circles: MD.

**Figure 8:**
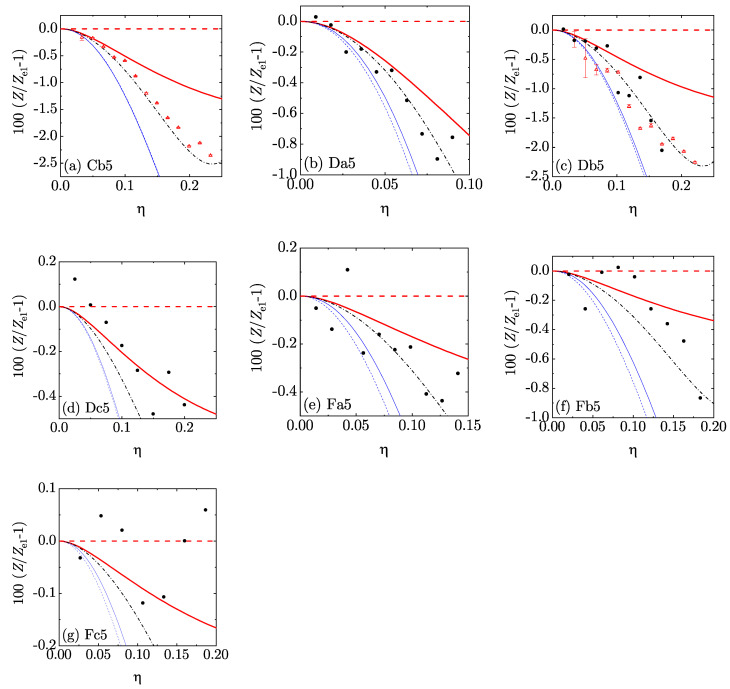
Plot of the relative deviations $100[Z\left(\eta \right)/Z_{e1}\left(\eta \right)-1]$ from the theoretical EOS $Z_{e1}\left(\eta \right)$ for the five-dimensional mixtures Cb5–Fc5 (see Table 2). Thick (red) dashed lines: e1; thick (red) solid lines: $\bar{e}$1; thin (blue) dashed lines: e2; thin (blue) solid lines: $\bar{e}$2; dash-dotted (black) lines: sp; filled (black) circles: MD; open (red) triangles with error bars in panels (**a**,**c**): MC.

In order to perform a more stringent assessment of the five theoretical EOS, we chose $Z_{e1}\left(\eta \right)$ as a *reference* theory and focused on the percentage deviation $100[Z\left(\eta \right)/Z_{e1}\left(\eta \right)-1]$ from it. The results are displayed in Figure 5 and Figure 6 for d=4 and Figure 7 and Figure 8 for d=5. Those figures reinforce the view that all our theoretical proposals are rather accurate: the errors in $Z_{e1}$ were typically smaller than 1% and they are even smaller in the other approximate EOS. Note that we have not put error bars in the MD data since they were unfortunately not reported in Reference [36]. We must also mention that the MD data were generally more scattered than the MC ones. Moreover, certain (small) discrepancies between MC and MD points can be observed in Figure 6c, MC data generally lying below MD data. The same feature is also present (although somewhat less apparent) in Figure 8c. This may be due to larger finite-size effects in the MD simulations than in the MC simulations: the MD simulations used 648 hyperspheres for d=4 and 512 or 1024 hyperspheres for d=5, while the MC simulations used 10,000 hyperspheres for d=4 and 3888 or 7776 for d=5. In any case, since the MC data were statistically precise, the discrepancy might be eliminated by the inclusion of the (unknown) error bars in the MD results. It is also worth pointing out that the representation of Figure 5, Figure 6, Figure 7 and Figure 8 is much more demanding than a conventional representation of *Z* vs. η for each mixture or even the representation of Figure 4.

## 4. Discussion and Concluding Remarks

In this paper we have carried out a thorough comparison between our theoretical proposals for the EOS of a multicomponent *d*-dimensional mixture of hard hyperspheres and the available simulation results for binary mixtures of both four- and five-dimensional hard hyperspheres. It should be stressed that in this comparison we have restricted ourselves to the liquid branch. Let us now summarize the outcome of the different theories for the compressibility factor.

First, we note that $Z_{\bar{e}2}\left(\eta \right)\approx Z_{e2}\left(\eta \right)<Z_{\mathrm{sp}}\left(\eta \right)<Z_{\bar{e}1}\left(\eta \right)<Z_{e1}\left(\eta \right)$. The fact that $Z_{\bar{e}2}\left(\eta \right)\approx Z_{e2}\left(\eta \right)$ is a consequence of the small deviations of $B_{3}^{e2}$ from the exact third virial coefficient (see Figure 2). Thus, there does not seem to be any practical advantage in choosing $Z_{\bar{e}2}$ instead of $Z_{e2}$, especially if d=4 [where the exact $B_{3}$ has a rather involved expression, see Equations (18)]. If one restricts oneself to the comparison between those approximate EOS that do not yield the exact $B_{3}$, namely $Z_{e1}$ and $Z_{e2}$, we find that $Z_{e2}$ performs generally better. On the other hand, if approximations requiring the exact $B_{3}$ as input are considered, namely $Z_{\bar{e}1}$, $Z_{\bar{e}2}$, and $Z_{\mathrm{sp}}$, the conclusion is that $Z_{\mathrm{sp}}$ generally outperforms the other two.

The comparison with the simulation data confirms that the good agreement between the results of $Z_{e1}\left(\eta \right)$ that had been found earlier in connection with both MD [36] and MC [57,59] simulation data are even improved by the other approximate theories. In fact, in both the four- and five-dimensional cases, the best agreement with the MD results is generally obtained from $Z_{\bar{e}1}$ and $Z_{\mathrm{sp}}$. On the other hand, for the four-dimensional case, the best agreement with the MC results corresponds to $Z_{\bar{e}2}\approx Z_{e2}$, while that for the five-dimensional case corresponds to $Z_{\mathrm{sp}}$.

Finally, it must be pointed out that it seems that overall $Z_{\mathrm{sp}}$ exhibits the best global behavior. However, more accurate simulation data would be needed to confirm this conclusion. It should also be stressed that the performance of the analyzed approximate EOS for fluid mixtures might be affected by the reliability of the (monocomponent) LM EOS. In any event, one may reasonably argue that the mapping between the compressibility factor of the mixture and the one of the monocomponent system with an effective packing fraction [see Equations (22) and (25)] that had already been tested in two- [64] and three-dimensional [93] mixtures is confirmed as an excellent approach also for higher dimensions.

## Figures and Tables

**Table 1:** Values of $b_{2}$–$b_{4}$, $\zeta_{0}$, $\zeta_{1}$, and $\eta_{\mathrm{cp}}$ for d=4 and 5.

	d=4	d=5
$b_{2}$	8	16
$b_{3}$	$2^{6}\left(\frac{4}{3}-\frac{3\sqrt{3}}{2\pi }\right)≃32.406$	106
$b_{4}$	$2^{9}\left(2-\frac{27\sqrt{3}}{4\pi }+\frac{832}{45\pi^{2}}\right)≃77.7452$	$\frac{25\;315\;393}{8\;008}+\frac{3\;888\;425\sqrt{2}}{4\;004\pi }-\frac{67\;183\;425\arccos(1/3)}{8\;008\pi }≃311.183$
$\zeta_{0}$	1.2973(59)	1.074(16)
$\zeta_{1}$	−0.062(13)	0.163(45)
$\eta_{\mathrm{cp}}$	$\frac{\pi^{2}}{16}≃0.617$	$\frac{\pi^{2}\sqrt{2}}{30}≃0.465$

**Table 2:** Binary mixtures of four- and five-dimensional hard spheres studied through simulations (Monte Carlo—MC or molecular dynamics—MD) and the values of their coefficients ${\bar{B}}_{2}$ [see Equation (9)], ${\bar{B}}_{3}$ [see Equations (16)–(18)], λ, and α [see Equation (23)].

*d*	Label	$\sigma_{2}/\sigma_{1}$	$x_{1}$	Simulation Method	${\bar{B}}_{2}$	${\bar{B}}_{3}$	λ	α
4	Aa4	1/4	0.25	MD ^1^	3.85618	12.2253	1.28824	0.677138
	Ab4	1/4	0.50	MD ^1^	5.21595	18.8828	1.10923	0.741033
	Ac4	1/4	0.75	MD ^1^	6.60436	25.6326	1.03810	0.862800
	Ba4	1/3	0.25	MD ^1^	4.42857	14.4931	1.28470	0.808392
	Bb4	1/3	0.50	MD ^1^	5.56098	20.2530	1.11943	0.816497
	Bc4	1/3	0.75	MD ^1^	6.77049	26.2935	1.04334	0.897356
	Cb4	2/5	0.50	MC ^2^	5.87285	21.5939	1.11692	0.868418
	Da4	1/2	0.25	MD ^1^	5.82895	20.8444	1.17876	0.958523
	Db4	1/2	0.50	MD ^1^ and MC ^2^	6.38235	23.9444	1.09883	0.928396
	Dc4	1/2	0.75	MD ^1^	7.15816	28.0333	1.04047	0.952376
	Eb4	3/5	0.50	MC ^2^	6.90085	26.5045	1.07078	0.966532
	Fa4	3/4	0.25	MD ^1^	7.55661	29.9061	1.03231	0.998173
	Fb4	3/4	0.50	MD ^1^	7.56231	29.9832	1.02894	0.992515
	Fc4	3/4	0.75	MD ^1^	7.73940	30.9790	1.01561	0.993060
5	Aa5	1/4	0.25	MD ^1^	6.30550	32.9426	1.24358	0.546995
	Ab5	1/4	0.50	MD ^1^	9.52439	57.2455	1.08739	0.671954
	Ac5	1/4	0.75	MD ^1^	12.7601	81.6145	1.02988	0.831562
	Ba5	1/3	0.25	MD ^1^	7.21951	37.7995	1.27656	0.675687
	Bb5	1/3	0.50	MD ^1^	10.0984	60.3097	1.10651	0.742645
	Bc5	1/3	0.75	MD ^1^	13.0411	83.1175	1.03739	0.863898
	Cb5	2/5	0.50	MC ^3,4^	10.6565	63.6666	1.11369	0.798464
	Da5	1/2	0.25	MD ^1^	9.89286	55.1378	1.22316	0.886983
	Db5	1/2	0.50	MD ^1^ and MC ^3,5^	11.6818	70.5615	1.10812	0.874437
	Dc5	1/2	0.75	MD ^1^	13.7964	88.0120	1.04172	0.925768
	Fa5	3/4	0.25	MD ^1^	14.5176	92.4875	1.04866	0.990981
	Fb5	3/4	0.50	MD ^1^	14.6327	93.8346	1.03957	0.982162
	Fc5	3/4	0.75	MD ^1^	15.2162	99.1168	1.02005	0.986104

${}^{1}$ Ref. [36], ${}^{2}$ Ref. [57], ${}^{3}$ Ref. [59], ${}^{4}$$x_{1}=\frac{971}{1944}=0.499486$, ${}^{5}$$x_{1}=\frac{973}{1944}=0.500514$.

## Abbreviations

The following abbreviations are used in this manuscript:

EOS	Equation of state
LM	Luban–Michels
MC	Monte Carlo
MD	Molecular dynamics
